# Antibiofilm efficacy of photoactivated curcumin, triple and double antibiotic paste, 2% chlorhexidine and calcium hydroxide against *Enterococcus fecalis in vitro*

**DOI:** 10.1038/srep24797

**Published:** 2016-04-21

**Authors:** Sharmila Devaraj, Nithya Jagannathan, Prasanna Neelakantan

**Affiliations:** 1 Biofilm Research Cluster, Saveetha Dental College and Hospitals, Saveetha University, Chennai, India; 2 Department of Oral and Maxillofacial Pathology. Saveetha Dental College and Hospitals, Saveetha University, Chennai, India; 3 Department of Conservative Dentistry and Endodontics, Saveetha Dental College and Hospitals, Saveetha University, Chennai, India

## Abstract

Root canal disinfection is one of the most important factors governing success of root canal treatment, especially when regenerative strategies are used. This study evaluated the efficacy of 5 intracanal medicaments against mature biofilms of *Enterococcus fecalis in vitro*: Light activated curcumin, triple antibiotic paste (TAP), double antibiotic paste (DAP), chlorhexidine, calcium hydroxide. Untreated teeth with biofilms served as controls. Confocal microscopy was used to analyse the biofilm mass and percentage of live/dead bacteria within the root canal as well as dentinal tubules. Dentinal shavings obtained from the root canal walls (at 200 and 400 microns depth) were used to quantify the colony forming units/mL. The results showed that light activated curcumin and triple antibiotic paste brought about complete disruption of the biofilm structure (P < 0.05) while chlorhexidine and calcium hydroxide were not significantly different from the control (P > 0.05). Light activated curcumin brought about the highest percentage of dead cells at both depths, but this was not significantly different from triple antibiotic paste (P > 0.05). Curcumin, TAP and DAP brought about a significant reduction of CFU/mL at both depths compared to the control and other groups (P < 0.05). Light activated curcumin brought about a 7 log reduction of bacteria at both depths.

Bacteria within root canal systems form highly organized communities called biofilms, which are highly resistant to chemomechanical debridement procedures^1^. Bacteria within the anatomical complexities of the root canal system (lateral and accessory canals, isthmi, apical deltas) may not be completely eliminated following biomechanics preparation of the root canal system. Effective elimination of such microbiota is essential for apical repair of mature and immature teeth^2^,^3^. The use of intracanal medicaments between appointments is therefore recommended to reduce the bacteria within root canal systems.

In the case of immature teeth with pulp necrosis, root canal disinfection is essential to aid in pulp revascularisation. While sodium hypochlorite continues to be the mainstay of root canal treatment as an irrigating solution, several intracanal medicaments have been proposed for these decontamination protocols^4^,^5^,^6^. The most commonly employed agents are triple antibiotic paste (a combination of metronidazole, ciprofloxacin and minocycline) or double antibiotic paste (a combination of metronidazole and ciprofloxacin)^6^,^7^. However recent reports suggest that these intracanal medicaments, including calcium hydroxide, triple antibiotic paste (TAP) and double antibiotic paste (DAP) could be potentially toxic to the cells of the apical papilla^8^ in addition to having deleterious effects on root dentin^9^.

An alternative technique of root canal disinfection involves photodynamic therapy (PDT). This method is less toxic to mammalian cells and less damaging to the dentin ultrastructure^10^. A recent report showed that light activated curcumin showed superior antibacterial activity than sodium hypochlorite, when used against *Enterococcus fecalis* biofilms, when used as a root canal irrigant^11^. However, it is not known if the antibacterial activity of photo activated curcumin is superior to that of other medicaments used in pulp revascularisation. This study was undertaken to compare the antibiofilm activity of photoactivated curcumin with triple antibiotic paste, double antibiotic paste, chlorhexidine and calcium hydroxide when used as an intracanal medicament.

## Materials and Methods

Single-rooted mandibular premolars with a closed apex [n = 110] were used in the study based on a study protocol that was approved by the Institutional Review Board and Ethics Committee of the University. These teeth were extracted for orthodontic reasons and informed consent was obtained from the patients. All experiments were performed in accordance with the appropriate guidelines. The teeth were collected in 0.01% sodium hypochlorite solution and maintained hydrated until use. The specimens were were prepared based on a previously established protocol described by Haapasalo and Orstavik^12^. Briefly, crowns (2–3 mm from the cement-enamel junction) and 3 to 5 mm of the apical portion of the root were removed to obtain specimens of 8 mm length. The cementum was removed after which the specimens were stored in saline until experimental procedures were performed. The cylindrical root sections were prepared using Gates-Glidden drills size 2 and 3 using 3% sodium hypochlorite as the irrigant. Smear layer was removed by placing the specimens in an ultrasonic bath of 5.25% sodium hypochlorite and 17% ethylene diamine tetraacetic acid for 4 min each. Following this, the specimens were rinsed in sterile water for 1 min and autoclaved (20 min at 121 °C). This approach was standardized following a pilot study which ensured sterility of the samples and is based on previously published reports^12^,^13^.

### Bacterial inoculation and biofilm generation

All microbiological procedures ensured strict adherence to aseptic techniques in a laminar flow cabinet. *E. fecalis* [ATCC 29212] was plated on sterile brain heart infusion broth supplemented with 1.5% [wt/vol] agar and incubated anaerobically at 37 °C for 24 h. A single colony of *E. fecalis* was collected from the agar plate and suspended in sterile brain heart infusion broth. Root specimens were placed in sterile centrifuge tubes containing 3 mL *E. fecalis* suspension [1 × 10^8^ mL^−1^] and incubated under anaerobic conditions at 37 °C for 4 weeks. This was done to obtain a mature biofilm of *E. fecalis*. Purity of culture was established by routine microbiological methods performed every week for the 4 week period. Fresh broth was replaced every second day to remove dead cells and to ensure bacterial viability. After incubation, the specimens were removed from the tubes aseptically and rinsed with sterile phosphate-buffered saline to remove the culture medium and non-adherent bacteria.

### Treatment of biofilms

Samples (n = 95) were irrigated with 3% NaOCl for 15 minutes followed by sterile saline (2 minutes), dried with paper points and divided into five groups (n = 19). Control specimens (n = 10) were retained in the incubator for the entire duration of the experiment. The root canals were filled with one of the following intracanal medicaments: group 1, curcumin (2.5 mg/ml of polyethylene glycol) activated with blue light (Bluephase LED, Ivoclar Vivadent, Liechtenstein;wavelength of 380–515 nm, intensity of 1200 mW/cm^2^ for 4 min), which was kept at a standardized distance of 1 mm from the root canal orifice; group 2, triple antibiotic paste (1 mg United States Pharmacopeia grade antibiotic powder compounded of equal portions of metronidazole, ciprofloxacin, and minocycline mixed with 1 mL sterile water at room temperature; group 3, double antibiotic paste (1 mg United States Pharmacopeia grade antibiotic powder compounded of equal portions of metronidazole and ciprofloxacin mixed with 1 mL sterile water), group 4, 2% chlorhexidine gel (Clorexoral gel 2%, Biodinamica, Ibipora, Brazil); group 5, calcium hydroxide gel (ApexCal, Ivoclar Vivadent, Liechtenstein). Each dressing was applied under sterile conditions via a syringe and needle until the dressing extruded from the apical opening.

After the excess material was removed, coronal and apical openings were sealed with temporary restorative cement (Cavit G, 3 M ESPE, AG, Seefeld, Germany). Then, specimens were placed into Petri dishes and covered with humid sterile gauzes and incubated at 37 °C for 14 days. All procedures were performed at room temperature under aseptic conditions by the same operator.

### Preparation of specimens for microbiological analysis

At the end of the 14 days period, the root canal was washed with 5 mL of sterile saline. Following this, the root canals were irrigated with 5 mL of the appropriate neutralising solution: neutralizing broth (Hi Media Labs, Bangalore, India) for curcumin, TAP and DAP, 0.5% Tween 80 (Sigma-Aldrich; Saint Louis, MO) in 0.07% soy lecithin for 2% chlorhexidine gel, 0.5% citric acid for calcium hydroxide. Root canals were flushed with sterile saline and dried with sterile paper points.

### Confocal laser scanning microscopic examination

Root sections from each group [n = 8] were stained with fluorescent LIVE/DEAD BacLight Bacterial Viability stain [Molecular Probes, Eugene, OR, USA] and viewed using a confocal laser scanning microscope [LSM 510, Carl Zeiss, Jena, Germany]. Six random areas of the biofilm on each dentin section were scanned with a 2 μm step size. Simultaneous dual-channel imaging was used to display the green and red fluorescence. Three dimensional reconstruction was done using OsiriX Imaging Software (Geneva, Switzerland), and quantification of the CLSM images was done using the BioimageL software^14^,^15^. The ratio of dead cells [red fluorescence to total fluoresence ratio] was calculated. Furthermore, the amount of dead bacteria was quantified at two regions of interest [200 and 400 microns] from the pulp-dentin junction toward the cementum. Statistical analysis of the data was done using one-way analysis of variance and Student-Newman-Keuls test. The alpha error was set at P = 0.05.

### Quantitative assessment of viable biofilm bacteria

Dentin debris from the root samples [n = 8] was harvested at 2 depths (200 and 400 microns) using Gates Glidden drills nos 4 and 5 [Mani Inc., Tochigi-Ken, Japan], respectively^16^, and collected in 1 mL of sterile brain heart infusion broth and incubated in an anaerobic environment at 37 °C for 24 h. The content of each microcentrifuge tube was serially diluted, 100 μL of broth in 100 μL of normal saline for 5 times. Five microliters of this sample was plated on brain heart infusion agar plates and incubated for 24 h. The microbial colony-forming units count [CFU/mL] was counted and the data were statistically analyzed with one-way ANOVA followed by Tukey multiple comparison [P = 0.05].

## Results

### Biofilm structure

**Figure 1:**
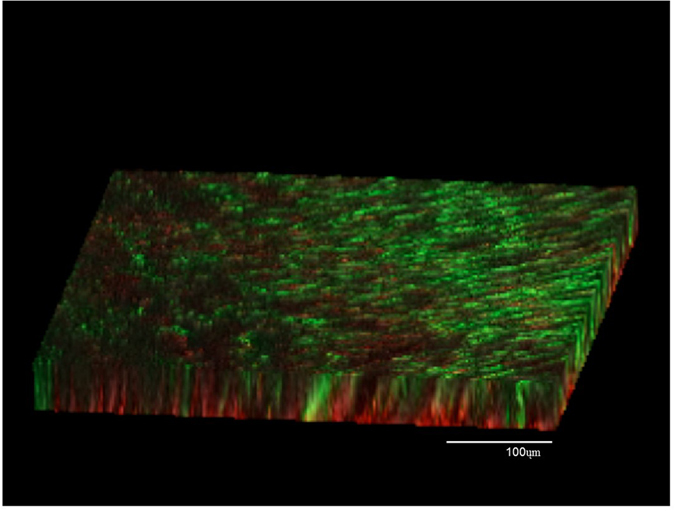
Three-dimensional reconstruction of confocal laser scanning microscopic images of *E. fecalis* biofilms in the control group. Note the high proportion of green channel [live bacteria].

The control specimens showed a dense multilayered biofilm with both live (green) and dead (red) cells, with a higher proportion of live cells (Fig. 1). The average thickness of the 4 week old biofilm in the control specimens was about 106 ± 11.4 μm. The biofilm architecture in the root canal lumen was completely disrupted in the specimens treated with curcumin (group 1) and TAP (group 2), while in was altered in group 3. The biofilm structure was not disturbed in groups 4 and 5. The thickness of the biofilm in groups 4 (88 ± 10.3 μm) and 5 (94 ± 9.7 μm) were not significantly different from the control, but was significantly higher than groups 1 and 2. The biofilm thickness in group 3 (DAP) decreased significantly when compared to the control (P < 0.05).

Considering the distribution of live/dead cells in the overall biomass, the percentage of red cells (dead bacteria) increased significantly in groups 1, 2 and 3 compared to the control and groups 4, 5 (P < 0.05). There was no significant difference in the percentage of dead cells amongst groups 1, 2 and 3 (P > 0.05).

### Intratubular biofilm

**Figure 2:**
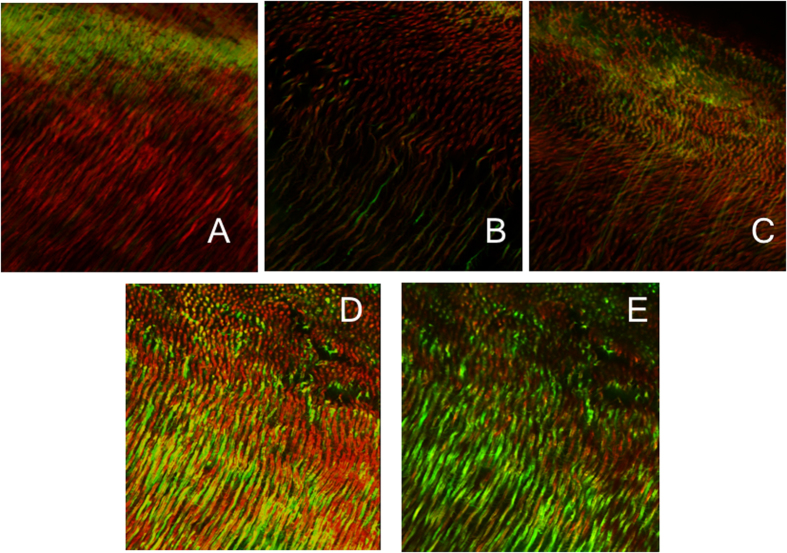
Three-dimensional reconstructions of confocal laser scanning microscopic images of *E. fecalis* biofilms. Images (**A–E**) show the biofilm after treatment with the experimental groups: (**A**) [photoactivated curcumin], (**B**) [triple antibiotic paste], (**C**) [double antibiotic paste], (**D**) [chlorhexidine], (**E**) [calcium hydroxide]. Note the high proportion of red channel [dead bacteria] in the group treated with light activated curcumin (**A**) and high proportion of green channel in the group treated with calcium hydroxide.

Representative CLSM images of bacteria within the dentinal tubules in the different groups has been presented (Fig. 2A–E). The highest percentage of dead bacteria within the dentinal tubules was found in group 1 (photoactivated curcumin) at both 200 microns and 400 microns. This was significantly higher than all other groups (P < 0.05), except group 2 (TAP) (P > 0.05). The percentage of dead bacteria in group 4 (CHX) was significantly higher than the control and group 5 (CH) at 200 microns (P < 0.05) while there was no significant difference between these groups at 400 microns (P > 0.05). At both depths, the percentage of dead bacteria was significantly higher in group 2 (TAP) than in group 3 (DAP) (P < 0.05).

### Quantitative assessment of viable biofilm bacteria

There was a significant reduction of viable bacteria in groups 1–3 compared to the control [P < 0.05]. At 200 microns depth, groups 1 and 2 showed a 7 log reduction of bacteria [no growth, P > 0.05]. Group 3 showed 4 log reduction of bacteria at 200 microns. This was significantly higher than groups 4 and 5 (P < 0.05). At 400 microns depth, group 1 (photoactivated curcumin) showed 7 log reduction of bacteria, followed by group 2 which showed a 6 log bacterial reduction (P > 0.05). There was no difference in the CFU/mL between groups 2 and 3 at 400 microns (P > 0.05). The CFU/mL in groups 4 and 5 were not significantly different from the control at both depths (P > 0.05). The results of the above analyses have been tabulated (Table 1).

## Discussion

The root canal biofilm is a complex structure composed of polysaccharide, protein and microbial cells. Pulp and periodical pathoses are host responses to biofilm mediated infection^17^. The procedure of regeneration has received considerable interest in endodontics recently. Such a protocol requires elimination of infection and disruption of the microbial ecosystem without or with minimal use of instrumentation^7^. Disinfection strategies in root canal treatment should be designed to eliminate the microbial reservoirs that persist within the dentinal tubules. Hence, in addition to antibacterial activity, it is imperative that these agents should be able to disrupt the bacterial biofilm^18^.

Intracanal medicaments used in regenerative endodontic procedures must not be toxic to mammalian cells, specifically stem cells of the apical papilla (SCAP)^7^,^19^. Earlier reports recommended the use of 1000 mg/mL of TAP and DAP prior to regenerative endodontic procedures to enable adequate root canal disinfection^7^. However, it has been shown that the toxicity of TAP and DAP to SCAP is concentration dependent with 1 mg/mL showing about 33–56% survival. Hence, it is presently recommended that 1 mg/mL of TAP and DAP be used as intracanal medicaments^8^. While calcium hydroxide has been shown to promote cell survival, its inability to act against *E. fecalis* has been well documented^8^,^20^. Similarly, CHX has been shown to be toxic to dental pulp stem cells^21^ and reports on its antibiofilm activity are inconclusive^22^,^23^.

This study was designed to offer two-fold data. Confocal microscopy provided information on the biofilm structure and percentage killing of bacteria within the biofilm. Furthermore, it was possible to identify isolated differences based on the depth of dentinal tubules in a three dimensional fashion^24^. The use of dentin powder helped provide quantitative data on the number of CFU/mL^16^. However, there is no conclusive evidence that this method offers adequate data on the degree of bacterial reduction within dentinal tubules^18^,^24^. The results of dentin powder analysis were in accordance with the results of the confocal microscopic analysis of percentage of live/dead bacteria.

This study tested the antibiofilm activity of intracanal medicaments both within the root canal lumen as well as dentinal tubules at two depths. The results showed that photo activated curcumin was as effective as TAP against *E. fecalis*. Both these agents were able to completely disrupt root canal biofilms compared to the control. Hence, the null hypothesis needs to be rejected. PDT involves the use of a photosensitising agent (light activated chemical), which can be preferentially localised in certain tissues. This is followed by irradiation with visible light (380–780 nm) to produce a series of events that lead to the generation of singlet oxygen and free radicals, which are cytotoxic to the microbial cells^25^. Several studies have shown that PDT is effective in root canal disinfection, without any untoward effects on mammalian cells and the dentin matrix. This could serve to be advantageous in regenerative endodontic strategies because of the weak nature of immature roots^26^. Another advantage of using curcumin as a photosensitiser is that it does not require the use of special light sources in contrast to conventional photosensitises such as methylene blue or toluene blue^27^.

The efficiency of curcumin against gram positive and gram negative bacteria has been demonstrated before^11^,^28^. The concentration of curcumin used was of 2.5 mg/mL based on a previously published report demonstrating the Minimum Inhibitory Concentration of curcumin at 625 μg/mL and minimum bactericidal concentration as 2.5 mg/mL^13^. The efficacy of photosensitiers depends on the concentration, radiant exposure and nature of the pharmaceutical preparation^28^. Specifically, it has been shown that curcumin demonstrated maximum antibacterial activity and stability when mixed with 5% aqueous polyethylene glycol (PEG) and maintained at a pH of 6.0^28^. Hence this study used curcumin mixed with 5% PEG. This concentration of PEG is important because increase in concentration of PEG decreases the antibacterial activity^29^.

For PDT to be effective, the photosensitiser must bind to the outer membrane of the target cell^30^. The superior antibiofilm activity of curcumin at the two depths evaluated in this study shows that this material is able to penetrate into the dentinal tubules to a depth of 400 microns. However, a major advantage of curcumin as a photosensitier is that it can exhibit lethal effects to microbial cells without binding or being in close proximity to the bacteria^28^. This may be attributed to the formation of hydrogen peroxide as an intermediary compound on light activation of curcumin.

TAP has been shown to be effective against *E. fecalis*^31^. The present study showed that TAP was able to completely disrupt the biofilm structure while DAP was unable to. However, the biofilm was structurally altered in specimens treated with DAP. Within the dentinal tubules, DAP killed less bacteria than TAP and curcumin. Since, no study has evaluated the dentinal tubule disinfection of DAP using CLSM, a direct comparison of results cannot be made. Sabrah *et al.* reported that TAP and DAP were similar in reduction of *E. fecalis* biofilms^32^. This difference from the results obtained in the present study could be attributed to methodological variables.

This study also evaluated the antibiofilm efficacy of CHX and CH. Although CH is able to penetrate to depths of 200 microns, it is ineffective against *E. fecalis* biofilms. This is in accordance with previous reports^33^. It has been reported that CHX exerts its antibacterial activity by disintegrating the cytoplasmic membranes and producing reactive oxygen species^34^. The results of the present study showed that neither CHX nor CH were able to destroy the biofilm structure. This is in agreement with published reports^22^,^33^. However, these results are in contrast to those of Baca *et al.*, who reported 100% biofilm inhibition^35^. This variation in result could be because of difference in the age of biofilms as well as the evaluation method. Furthermore, CHX is extremely toxic to SCAP and hence not recommended as an intracanal medicament in regenerative endodontic procedures^21^.

To the best knowledge of the authors, this appears to be the first study to compare the activity of photoactivated curcumin with currently recommended intracanal medicaments (triple antibiotic paste, double antibiotic paste, CH and CHX) against mature biofilms of *E. fecalis*. As discussed earlier, TAP, DAP and CHX are toxic to the SCAP when used as intracanal medicaments in regenerative endodontic treatment protocols. Furthermore, CH and CHX when used as intracanal medicaments, are unable to disrupt *E. fecalis* biofilms^36^,^37^. Since photoactivated disinfection brings about instantaneous killing of bacteria, it is highly unlikely for resistant strains to develop, in contrast to the use of antibiotics, which is a very important consideration to be made.

In conclusion, photoactivated curcumin demonstrated superior antibiofilm and antibacterial activity against *E. fecalis* than triple antibiotic paste, but the difference was not statistically significant. While chlorhexidine killed more *E. fecalis* cells than calcium hydroxide within the dentinal tubules at 200 microns depth, both these agents were ineffective in disrupting the structure of *E. fecalis* biofilms. Future studies should evaluate the cytotoxic effects of curcumin against stem cells to recommend its use as an intracanal medicament in regenerative endodontic procedures.

## Additional Information

**How to cite this article**: Devaraj, S. *et al.* Antibiofilm efficacy of photoactivated curcumin, triple and double antibiotic paste, 2% chlorhexidine and calcium hydroxide against *Enterococcus fecalis in vitro. Sci. Rep.* **6**, 24797; doi: 10.1038/srep24797 (2016).

## Figures and Tables

**Table 1:** Biofilm thickness, percent of apparently dead bacterial cells in the overall biofilm mass and bacterial colony-forming units (CFU/mL) within the dentinal tubules at 200 and 400 microns depth, assessed by confocal laser microscopy and microbial culture analysis after different treatment regimes.

Group	Biofilm Thickness (μm)	Dead Bacteria in Biofilm Mass (%)	Dead Cells % at 200 Microns Depth	Dead Cells% at 400 Microns Depth	CFU/mL at 200 microns	CFU/mL at 400 microns
Photoactivated curcumin (Group 1)	2.863 ± 0.64^a^	98.12 ± 2.7^a^	98.93 ± 4.17^a^	93.84 ± 4.73^a^	1.1 × 10^2^ ± 0.12 × 10^2a^	3.7 × 10^2^ ± 0.84 × 10^2a^
Triple antibiotic paste (Group 2)	4.14 ± 1.02^a^	93.3 ± 1.42^a^	92.64 ± 5.18^a^	81.15 ± 3.28^a^	3.2 × 10^2^ ± 0.84 × 10^2a^	4.7 × 10^3^ ± 0.91 × 10^2a^
Double antibiotic paste (Group 3)	21.74 ± 3.67^b^	83.25 ± 6.17^b^	70.62 ± 4.71^b^	55.81 ± 4.54^b^	2.6 × 10^5^ ± 0.72 × 10^5b^	6.14 × 10^3^ ± 0.65 × 10^2a^
Chlorhexidine (Group 4)	88 ± 10.3^c^	4.1 ± 1.3^c^	24.44 ± 3.29^c^	1.2 ± 0.26^c^	1.1 × 10^9^ ± 0.36 × 10^9c^	4.1 × 10^9^ ± 0.82 × 10^9b^
Calcium hydroxide (Group 5)	94 ± 9.7^c^	2.1 ± 0.22^c^	4.12 ± 1.97^d^	1.12 ± 0.17^c^	1.9 × 10^9^ ± 0.61 × 10^9c^	3.9 × 10^9^ ± 0.33 × 10^9b^
Control	106 ± 11.4^c^	1.94 ± 0.15^c^	1.02 ± 1.3^d^	0.96 ± 0.04^c^	2.3 × 10^9^ ± 0.42 × 10^9c^	4.8 × 10^9^ ± 0.66 × 10^9b^

Within each column, values with identical lower case superscript alphabet indicates no significant difference (P  >  0.05).
